# First Trimester Ultrasound in Prenatal Diagnosis—Part of the Turning Pyramid of Prenatal Care

**DOI:** 10.3390/jcm3030986

**Published:** 2014-09-05

**Authors:** Ran Neiger

**Affiliations:** Director of the Maternal-Fetal Medicine Unit, Ma’ayanei Hayeshua Hospital, Bnei Brak 51544, Israel; E-Mail: ran@neiger.com; Tel.: +972-54-5657191.

**Keywords:** first trimester sonography, nuchal translucency, chromosomal aneuploidies, fetal anomalies

## Abstract

First-trimester sonographic assessment of the risk of chromosomal abnormalities is routinely performed throughout the world, primarily by measuring fetal nuchal translucency thickness between 11–13 weeks’ gestation, combined with assessment of serum markers. The development of high-frequency transvaginal transducers has led to improved ultrasound resolution and better visualization of fetal anatomy during the first-trimester. Continuous improvement in ultrasound technology allows a thorough detailed assessment of fetal anatomy at the time of the nuchal translucency study. Using transabdominal or transvaginal sonography, or a combination of both approaches, it is now possible to diagnose a wide range of fetal anomalies during the first trimester. Multiple studies reported early diagnosis of major fetal anomalies after demonstrating the association of increased nuchal translucency thickness with structural defect in chromosomally normal and abnormal fetuses. Normal sonographic findings provide reassurance for women at high risk while detection of fetal malformation during the first trimester enables discussion and decisions about possible treatments and interventions, including termination of pregnancy, during an early stage of pregnancy.

First trimester sonography is used mainly for simple tasks that include assessment of fetal viability, confirmation of gestational age, establishment of chorionicity in multiple gestation, evaluation of pelvic masses and ruling out ectopic pregnancy. The value of real-time sonographic examination during the first trimester was recognized as soon as the technology became available [1], and the accuracy and diagnostic ability improved substantially with the introduction of transvaginal sonography shortly thereafter. The rapid improvement of imaging due to high frequency and high resolution achieved through advances in signal processing and image magnification contributed to a much greater use of first-trimester studies: It enabled first trimester assessment of fetal anatomy. Starting during early days of obstetric sonography, it became evident that normal and abnormal anatomy of the fetus could be visualized as never before. For example, while in 1972 the sonographic detection of a single case of anencephaly at 17 weeks’ gestation merited a publication in the *Lancet* [2], in 1987 Schmidt *et al.* were able to describe the sonographic visualization of physiologic anterior abdominal wall hernia at 8–9 weeks’ gestation and the return of the intestine into the abdominal cavity into the peritoneal cavity by 10–12 weeks [3]. The recognition of first trimester sonography ability and benefits led to a rapid increase in use over the subsequent years. In 1988, Green and Hobbins described the transabdominal ultrasound examination of the first trimester fetus [4]. However, it was the introduction of transvaginal sonography that revolutionized first-trimester obstetric sonography. A succession of major breakthroughs was achieved within a short period. Rottem *et al.* published their experience in first trimester transvaginal sonographic diagnosis of fetal anomalies in 1989 [5], and in 1990 there were several publications regarding transvaginal sonographic detection of cardiac malformations during the first trimester [6,7]. Continuous technologic improvements and the growing recognition of sonographic appearance of normal and abnormal anatomy enabled improved detection of congenital malformations and genetic disorders. In 1991, Dolkar and Reimers published normative data for transvaginal fetal echocardiography in early pregnancy [8]; in the same year, D’Amelio *et al.* published a comparative study on performing fetal echocardiography during early pregnancy [9] and Bronshtein *et al.* described their experience in first-trimester detection of fetal cardiac abnormalities [10].

A turning point in first-trimester sonography occurred in 1992 when Nicolaides *et al.* reported that the incidence of chromosomal defects was 35% among 51 fetuses with nuchal translucency ≥3 mm, compared with 1% in 776 fetuses with thinner nuchal translucency [11]. Multiple studies subsequently confirmed the significance of this first-trimester sonographic marker. Sonographic technology that included high-frequency transvaginal probes and color Doppler flow imaging enabled researchers to conduct detailed first-trimester investigation of fetal anatomy and hemodynamics. It enabled the detection of findings such as reverse diastolic umbilical artery flow in the first trimester which was later found to be associated with chromosomal fetal abnormalities and cardiac defects [12,13,14]. In 1994 Achiron *et al.* described first-trimester diagnosis of fetal congenital heart disease by transvaginal sonography [15]. Using high-resolution transvaginal sonography these researchers were able to detect conditions such as ectopia cordis with ventricular septal defect, ventricular septal defect with persistent truncus arteriosus, tetralogy of Fallot, *etc.* Associated abnormalities detected sonographically included omphalocele, bilateral renal agenesis and more. The authors concluded that the advances in transvaginal sonographic technology opened a new era in the evaluation of fetal development during the early stages of gestation [15].

Following these sonographic revelations, progress in the field of first trimester sonography continued in two intertwined directions: One was finding additional sonographic markers that increased the accuracy and sensitivity of detecting chromosomal abnormalities, especially Down syndrome. The other was improving the early detection of congenital anomalies. However, there was no clear distinction between those who studied normal and abnormal fetal anatomy and the researchers who concentrated on first trimester sonographic screening for fetal aneuploidies [16]. Several publications regarding increased nuchal translucency (NT) in fetuses with trisomy 21 and in association with cardiac defects appeared in 1995 [17,18,19,20]. Pandya *et al.* studied 1015 pregnancies with increased fetal nuchal translucency and reported that the observed number of trisomies 21, 18 and 13 in fetuses with NT measurement of 3 mm, 4 mm, 5 mm and >6 mm were approximately 3 times, 18 times, 28 times and 36 times higher than the expectant number by maternal age [17]. The incidences of Turner syndrome and triploidy were 9 times and 8 times higher than in unselected population of 5814 women undergoing first-trimester fetal karyotyping for maternal age [17]. In the same journal, this group also reported that the implementation of first trimester measurement of nuchal translucency as part of routine prenatal care enabled the detection of three of the four fetuses with Down syndrome in their population [18]. In 1995, this group also published their experience in measuring NT in twin pregnancies [21]. In 1997, Souka and Nicolaides published a review article in which they described abnormalities diagnosed by the 10–14 weeks scan [22]. In addition to the multiple various organ-system defects they described (acrania, exencephaly, anencephaly, encephalocele, hydrocephalus and holoprosencephally were just several of the cranial defects they described), they reviewed the significance of fetal nuchal translucency measurement: This marker provided an effective method of screening for chromosomal abnormalities and major cardiac defects since increased nuchal translucency was commonly associated with a wide range of fetal abnormalities and genetic syndromes. The clear benefits and the ease of ascertainment of nuchal translucency screening led Nicolaides’ group to become a leading force that continuously broadened the use of universal nuchal translucency screening [23]. In 1998, they published their experience in first-trimester measurement of nuchal translucency for detection of fetuses with sex chromosome abnormalities [24] and the following year the use of nuchal translucency measurement for detection of trisomy 13 [25]. The same year, Maymon *et al.* published their assessment of the feasibility of first-trimester nuchal translucency measurement in higher order multiple gestations [26]. In 2001, Cicero *et al.* reported that by studying the fetal profile at the 11–14 weeks scan they determined that the nasal bone was not visible in 73% of fetuses with trisomy 21 [27]. Two years later this group suggested that by integrating the nasal bone measurement with nuchal translucency and the first-trimester serum markers free beta-human chorionic gonadotropin (β-hCG) and pregnancy-associated plasma protein-A (PAPP-A) for a false-positive rate of 5%, the detection rate of Down syndrome would increase to 97% [28]. On the other side of the Atlantic Ocean, in 2002, Lorraine Dugoff reviewed the use of first-trimester ultrasound in prenatal diagnosis of fetal structural abnormalities (and genetic syndromes) in the central nervous system, renal, gastrointestinal, cardiac, skeletal and other organ systems [29].

The advantages of first trimester screening for early detection of abnormal pregnancies were clear to both pregnant women and their physicians. It decreased the need for invasive testing (previously offered to every 35 years old, thereby missing abnormal pregnancies in younger women) and the associated risk of miscarriages, and enabled first-trimester invasive testing and diagnosis in high-risk pregnancies using chorionic villi sampling rather than a second trimester amniocentesis. It also allowed the safer and more private option of first-trimester termination of an abnormal pregnancy before the pregnancy was visible, decreased anxiety and provided reassurance about pregnancy well-being in both high-risk and low-risk situations. Research into women’s attitude toward first- *versus* second-trimester screening showed an overwhelming enthusiasm for early screening; in a study published in 2000, 74% of the women interviewed chose first-trimester rather than second-trimester screening [30]. Sixty-nine percent of women stated they would choose to undergo first-trimester screening to learn whether their fetus had Down syndrome even if all abnormal pregnancies identified by this method resulted in miscarriage before the second trimester. These women felt there was a benefit in gaining the information provided by the screening in understanding the reason for the miscarriage if it occurred [30]. In another study, 97.8% of women preferred screening to be carried out in the first, rather than the second trimester [31].

In 2003, Kypros Nicolaides, who has been a leading force in research, education and implementation of first trimester screening for chromosomal defects, published an editorial regarding screening for chromosomal abnormalities [32]. At the time, methods of screening to identify pregnancies at high-risk for chromosomal abnormalities (those that would be offered invasive testing) were maternal age (= background risk), ultrasound findings at 11–14 weeks and/or in the second trimester and maternal serum biochemical testing at 11–14 weeks and/or in the second trimester. Nicolaides proposed that screening the population at 11–14 weeks by combining maternal age with sonographic measurement of fetal NT, assessment of the presence of nasal bone and maternal serum measurement of free β-hCG and PAPP-A would yield a detection rate of 95% with an invasive testing rate of about 2%. Second-trimester ultrasound study of fetal anatomy would detect >70% of trisomy 21 fetuses who had not been identified in the first trimester [32]. The following year Nicolaides published another extensive review article regarding first trimester sonographic markers of chromosomal abnormalities in which he summarized prospective studies of 200,868 pregnancies including 871 fetuses with trisomy 21 [33]. In addition to increased nuchal translucency, Nicolaides listed important first-trimester sonographic markers of chromosomal abnormalities that included absence of nasal bone, abnormal flow velocity patterns in the ductus venosus and maxillary hypoplasia in fetuses with trisomy 21, early onset fetal growth restriction, bradycardia, exomphalos, absent nasal bone and single umbilical artery in trisomy 18, tachycardia, early onset growth restriction, megacystis and holoprosencephaly or exomphalos in trisomy 13. In the same year, Heath and Nicolaides published a chapter on sonographic features of chromosomal defects in which they discussed the usefulness (or lack thereof) of additional first-trimester sonographic findings such as of short femur, humerus and ears in fetuses with trisomy 21, the presence of choroid plexus cysts, pyelectasis and cardiac echogenic foci, placental volume and Doppler studies of uterine artery, umbilical artery and vein, *etc.* [34].

Most researchers described assessment of the nasal bone using two-dimensional sonography [35]. Three-dimensional evaluation of mid-facial hypoplasia in fetuses with trisomy 21 at 11–13 weeks’ gestation was described by Nicolaides’ group in 2006 [36]. Meanwhile, additional sonographic markers were identified and added to first trimester fetal assessment in order to improve the detection rate of chromosomal abnormalities. In 2007, Sonek *et al.* described a method of determining the position of the front of the maxilla with respect to the fetal forehead (the frontomaxillary facial angle (FMF angle)) at 11–13 + 6 weeks’ gestation [37]. This angle was significantly larger in fetuses with trisomy 21 than in euploid fetuses. First-trimester studies of the FMF angle were also conducted in fetuses with trisomy 18 and 13 [38,39] and in fetuses with spina bifida [40]. Bernard *et al.* reported that, while in their opinion these early sonographic features may be too complex for routine screening, they had been successful in first trimester sonographic screening for fetal spinal bifida using fetal biparietal diameter (BPD) in 34,951 unselected consecutive pregnancies; the BPD of 22 of 44 fetuses with spina bifida (50%) was at the <5th percentile [41]. More recently, the use of FMF angle measurement was again found to increase the effectiveness of screening for trisomy 18 [42].

The First- and Second-Trimester Evaluation of Risk Research Consortium trial, the largest prospective US study of first-trimester risk assessment at that time, found that first-trimester risk assessment provided efficient Down syndrome risk assessment and was superior to the second-trimester serum screen [43]. In 2008, Reddy *et al.* published the commentary of a workshop held in 2006 by The Eunice Kennedy Shriver National Institute of Child Health and Human Development in order to summarize the available evidence on the role and performance of current fetal imaging technology, and to establish a research agenda [44]. They concluded that ultrasonography was the imaging modality of choice for pregnancy evaluation due to its relatively low cost, real-time capability, safety, and operator comfort and experience. First-trimester ultrasonography extended the available window for fetal evaluation and raised the possibility of performing early anatomic survey. First-trimester studies for the purpose of identifying abnormal fetal anatomy, hemodynamic problems and screening for genetic anomalies continued to be combined. For example, Faiola *et al.* successfully examined the tricuspid valve in 718 fetuses at the 11 to 13 + 6-week scan and calculated the likelihood ratio for trisomy 21 in fetuses with tricuspid regurgitation [45]. Kagan *et al.* studied NT in 512 monochorionic twin pregnancies at 11–13 + 6 weeks’ gestation and concluded that discordance in NT of 20% or more was found in about 25%; the risk of early fetal death or development of severe twin-to-twin transfusion syndrome (TTTS) in this group was more than 30% [46]. In 2008, Maize *et al.* published their first-trimester observations in predicting major fetal anomalies and fetal death in fetuses with abnormal flow in the ductus venosus [47]. They reported that although reversed a-wave was associated with increased risk for chromosomal abnormalities, cardiac defects and fetal death, in about 80% of cases with reversed a-wave the pregnancy outcome was normal. Bilardo *et al.* reported that finding low-resistance hepatic artery flow in the first trimester was associated with adverse outcome [48]. Despite the advances made in studying fetal anatomy during the first trimester, and while prenatal screening and diagnosis of trisomy 21 had shifted from the second trimester to the first trimester, routine assessment of fetal anatomy continued to be conducted mainly in the second trimester.

In 2011, Kypros Nicolaides introduced the concept of turning the pyramid of care in which he proposed that since many pregnancy complications could be predicted at an integrated visit at 11–13 weeks, the tradition of closely following pregnancies more frequently in the third trimester should be inverted, with the emphasis placed on the first, rather than the third trimester of pregnancy [49]. In that year he also published a comprehensive updated review of screening for fetal aneuploidies at 11–13 weeks [50]. In his review he stated that the effective first trimester screening for major aneuploidies by combination of fetal nuchal translucency and maternal serum markers, that could identify about 90% of affected fetuses, could be further improved by inclusion of assessment of the nasal bone and flow in the ductus venosus, hepatic artery and across the tricuspid valve. Assessment of each of these markers could be incorporated into first-trimester combined screening, increasing the detection rate to 93%–96% and decreasing the false-positive rate to 2.5%. He also recommended that the biochemical test be performed at 9–10 weeks and the ultrasound scan at 12 weeks [50].

Recent reports by different researchers consistently demonstrate the accuracy of first-trimester sonographic studies in detecting anatomical anomalies [51,52,53]. The current practice of first-trimester screening for genetic defects combined with assessment of fetal anatomy, which enable identification of perinatal problems and diagnosis of high-risk conditions, has been endorsed by leading researchers [54]. However, first trimester study of fetal anatomical structure has its limitations. For example, not all cardiac, skeletal, central nervous system and other organ system anomalies are visualized well in the first trimester. Other considerations of first trimester sonography are the necessary skills required of the examiner and the cost involved. High quality equipment and well trained providers are needed to enable adequate assessment of fetal anatomy in the first trimester. A subsequent second-trimester scan is often performed in order to detect additional anomalies, thus doubling the number of studies required. In 2009, Timor-Tritsch *et al.* published their commentary on fetal anatomy assessment at the time of first-trimester screening [55]. After reviewing the available literature they concluded that both normal and abnormal fetal anatomy can be detected between 11–14 weeks’ gestation. However, they pointed out the heterogenic nature of the studies of first-trimester anatomic surveys and suggested a collaborative research to systematically investigate the diagnostic capabilities of such studies [55]. The accuracy of early sonography in detection of fetal anomalies has recently been reviewed by Rossi and Prefumo [56]. In their review of the results of 19 studies that included a total of 78,002 fetuses that underwent ultrasound at 11–14 weeks’ gestation, there were 996 malformed fetuses. The detection rate was 51%. The highest detection rate was of neck anomalies (92%) and the lowest detection was of face, genitourinary and limb defects (34% each). The rate of detection of cardiac anomalies was 43% and increased to 53% when fetal echocardiography was performed. Multiple defects were identified more frequently than isolated malformations (60% compared with 44%, *p* = 0.005). The detection rate was higher (62%) when transabdominal sonography was combined with transvaginal sonography than when either approach was performed alone (51% and 34%, respectively). Detection rate was higher in women at high risk (65%) than unselected population (50%). The authors concluded that because of the late development of several organ systems, a number of fetal malformations remain undetected by early ultrasonography [56]. Another consideration is the maternal anxiety that could be caused by first-trimester sonographic findings that may subsequently spontaneously resolve, or findings that have no clinical significance. For example, Dukhovny *et al.* recently reported that after excluding aneuploidy pregnancies, they followed 57 fetuses with a sonographic appearance of absent nasal bone. Three euploid fetuses were found to have additional anomalies by second-trimester ultrasound study. According to the authors, for the other 54 fetuses, normal newborn examination findings provided “some reassurance especially in the setting of otherwise normal second-trimester sonographic findings” [57].

Currently, highly effective first-trimester sonographic assessment of the risk of chromosomal abnormalities is routinely performed throughout the world, primarily by measuring fetal nuchal translucency thickness between 11–13 weeks’ gestation combined with assessment of serum markers. Continuous improvement in ultrasound technology allows a thorough detailed assessment of fetal anatomy at the time of the nuchal translucency study. The development of high-frequency transvaginal transducers has led to improved ultrasound resolution and better visualization of fetal anatomy during the first-trimester. Additionally, researchers continuously investigate improved methods of first-trimester assessment [58]. During early anatomic study, the entire fetus fits in the transducer’s focal zone, the frequent changes of the fetal position enable evaluation from many directions and the position of the fetus can be changed by the examiner. In obese patients, early transvaginal sonography may be the only opportunity for an adequate structural evaluation. In addition, some anomalies are better seen before 14 weeks’ gestation because they regress later [44]. Multiple studies reported early diagnosis of major fetal anomalies after demonstrating the association of increased nuchal translucency thickness with structural defects in chromosomally normal and abnormal fetuses. Using transabdominal or transvaginal sonography, or a combination of both, it is now possible to diagnose a wide range of fetal anomalies during the first trimester. The recent introduction of non-invasive perinatal testing (NIPT) using cell-free fetal DNA has brought into question the value of routine use of first trimester ultrasound. Specifically, if NIPT became the primary screening test for all women would there be a need for the first trimester scan? The answer may be found in studies that assessed the performance of first trimester sonographic screening of chromosomally normal fetuses. In the 1995 study by Pandya *et al.* [17] the incidence of structural defects among 821 chromosomally normal fetuses was approximately 4% and included cardiac, diaphragmatic, renal and abdominal wall abnormalities. Survival decreased from 97% for those with nuchal translucency thickness of 3 mm to 91% for a nuchal translucency thickness of 4 mm and 53% for a nuchal translucency thickness of ≥5 mm. In a 2006 study of 39,572 unselected women by Saltvedt *et al.*, after excluding chromosomally abnormal pregnancies, the authors detected 69% of lethal anomalies by first trimester scan at 12–14 weeks’ [59]. Normal sonographic findings provide reassurance for women at high risk while detection of fetal malformation during first trimester enables discussion and decisions about possible treatments and interventions, including termination of pregnancy, during early stage of pregnancy.

## References

[1] Romero R., Jeanty P., Hobbins J.C. (1984). Diagnostic ultrasound in the first trimester of pregnancy. Clin. Obstet. Gynecol..

[2] Campell S., Holt E.M., Johnston F.D., May P. (1972). Anencephaly: Early ultrasound diagnosis and active management. Lancet.

[3] Schmidt W., Yarkoni S., Crelin E.S., Hobbins J.C. (1987). Sonographic visualization of physiologic anterior wall hernia in the first trimester. Obstet. Gynecol..

[4] Green J.J., Hobbins J.C. (1988). Abdominal ultrasound examination of the first trimester fetus. Am. J. Obstet. Gynecol..

[5] Rottem S., Bronshtein M., Thaler I., Brandes J.M. (1989). First trimester transvaginal sonographic diagnosis of fetal anomalies. Lancet.

[6] Bronshtein M., Sigler E., Yoffe N., Zimmer E.Z. (1990). Prenatal diagnosis of ventricular septal defect and overriding aorta at 14 weeks’ gestation, using transvaginal sonography. Prenat. Diagn..

[7] Gembruch U., Knopfle G., Chatterjee M., Bald R., Hansmann M. (1990). First-trimester diagnosis of fetal congenital heart disease by transvaginal two-dimentional and Doppler echocardiography. Obstet. Gynecol..

[8] Dolkart L.A., Reimers F.T. (1991). Transvaginal fetal echocardiography in early pregnancy: Normative data. Am. J. Obstet. Gynecol..

[9] D’Amelio R., Giorlandino C., Masala L., Garofalo M., Martinelli M., Anelli G., Zichella L. (1991). Fetal echocardiography using transvaginal and transabdominal probes during the first period period of pregnancy: A comprehensive study. Prenat. Diagn..

[10] Bronshtein M., Zimmer E.Z., Milo S., Ho S.Y., Lorber A., Gerlis L.M. (1991). Fetal cardiac abnormalities detected by transvaginal sonography at 12–16 weeks’ gestation. Obstet. Gynecol..

[11] Nicolaides K.H., Azar G., Byrne D., Mansur C., Marks K. (1992). Fetal nuchal translucency: Ultrasound screening for chromosomal defects in first trimester of pregnancy. BMJ.

[12] Ariyuki Y., Hata T., Kitao M. (1993). Reversed end-diastolic umbilical artery velocity in a case of intrauterine fetal death at 14 weeks’ gestation. Am. J. Obstet. Gynecol..

[13] Montenegro N., Beires J., Leite L.P. (1995). Reversed end-diastolic umbilical artery blood flow at 11 weeks’ gestation. Ultrasound Obstet. Gynecol..

[14] Murta C.G., Moron A.F., Avila M.P. (2000). Reversed diastolic umbilical artery flow in the first trimester associated with chromosomal fetal abnormalities or cardiac defects. Obstet. Gynecol..

[15] Achiron R., Rotstein Z., Lipitz S., Mashiach S., Hegesh J. (1994). First trimester diagnosis of fetal congenital heart disease by transvaginal ultrasonography. Obstet. Gynecol..

[16] Szabo J., Gallen J., Szemere G. (1995). First trimester ultrasound screening for fetal aneuploidied in women over 35 and under 35 years of age. Ultrasound Obstet. Gynecol..

[17] Pandya P.P., Kondylios A., Hibert L., Snijders R.J.M., Nicolaides K.H. (1995). Chromosomal defects and outcome in 1015 fetuses with increased nuchal translucency. Ultrasound Obetet. Gynecol..

[18] Pandya P.P., Goldberg H., Walton B., Riddle A., Shelley S., Snijders R.J., Nicolaides K.H. (1995). The implementation of first-trimester scanning at 10–13 weeks’ gestation and the measurement of fetal nuchal translucency thickness in two maternity units. Ultrasound Obstet. Gynecol..

[19] Hyett J.A., Moscoso G., Nicolaides K.H. (1995). First trimester nuchal translucency and cardiac septal defects in fetuses with trisomy 21. Am. J. Obstet. Gynecol..

[20] Hyett J.A., Moscoso G., Nicolaides K.H. (1995). Increased nuchal translucency in trisomy 21 fetuses: Relation to narrowing of the aortic isthmus. Hum. Reprod..

[21] Pandya P.P., Hilbert F., Snijders R.J., Nicolaides K.H. (1995). Nuchal translucency thickness and crown-rump length in twin pregnancies with chromosomally abnormal fetuses. J. Ultrasound Med..

[22] Souka A.P., Nicolaides K.H. (1997). Diagnosis of fetal abnormalities at the 10–14-week scan. Ultrasound Obstet. Gynecol..

[23] Snijders R.J.M., Noble P., Sebire N., Souka A., Nicolaides K.H. (1998). UK multicenter project on assessment of risk of trisomy 21 by maternal age and fetal nuchal translucency thickness at 10–14 weeks of gestation. Lancet.

[24] Sebire N.J., Snijders R.J., Brown R., Southall T., Nicolaides K.H. (1998). Detection of sex chromosome abnormalities by nuchal translucency screening at 10–14 weeks. Prenat. Diagn..

[25] Maymon R., Dreazen E., Tovbin Y., Bukovsky I., Weinraub Z., Herman A. (1999). The feasibility of nuchal translucency measurement in higher order multiple gestations achieved by assisted reproduction. Hum. Reprod..

[26] Snijders R.J.M., Sebire N.J., Nayar R., Souka A., Nicolaides K.H. (1999). Increased nuchal translucency in trisomy 13 fetuses at 10–14 weeks of gestation. Am. J. Med. Genet..

[27] Cicero S., Curcio P., Papageorghiou A., Sonek J., Nicolaides K.H. (2001). Asence of nasal bone in fetuses with trisomy 21 at 11–14 weeks of gestation: An observational study. Lancet.

[28] Cicero S., Bindra R., Rembouskos G., Spencer K., Nicolaides K.H. (2003). Integrated ultrasound and biochemical screening for trisomy 21 using fetal nuchal translucency, absent nasal bome, free β-hCG and PAPP-A at 11–14 weeks. Prenat. Diagn..

[29] Dugoff L. (2002). Ultrasound diagnosis of structural abnormalities in the first trimester. Prenat. Diagn..

[30] Mulvey S., Wallace E.M. (2000). Women’s knowledge of and attitude to first and second trimester screening for Down’s syndrome. BJOG.

[31] De Graaf I.M., Tijmstra T., Bleker O.P., van Lith J.M. (2002). Womens preference in Down syndrome screening. Prenat. Diagn..

[32] Nicolaides K.H. (2003). Screening for chromosomal abnormalities. Ultrasound Obstet. Gynecol..

[33] Nicolaides K.H. (2004). Nuchal translucency and other first-trimester sonographic markers of chromosomal abnormalities. Am. J. Obstet. Gynecol..

[34] Heath V., Nicolaides K. (2004). Sonographic features of chromosomal defects. The 11–13 + 6 Weeks Scan.

[35] Sonek J.D., Cicero S., Neiger R., Nicolaides K.H. (2006). Nasal bone assessment in prenatal screening for trisomy 21. Am. J. Obstet. Gynecol..

[36] Dagklis T., Borenstein M., Perelta C.F., Faro C., Nicolaides K.H. (2006). Three-dimensional evaluation of mid-facial hypoplasia in fetuses with trisomy 21 at 11 + 0 to 13 + 6 weeks of gestation. Ultrasound Obstet. Gynecol..

[37] Sonek J., Borenstein M., Dagklis T., Persico N., Nicolaides K. (2007). Frontomaxillary facial angle in fetuses with trisomy 21 at 11–13 + 6 weeks. Am. J. Obstet. Gynecol..

[38] Borenstein M., Persico N., Strobl I., Sonek J., Nicolaides K.H. (2007). Frontomaxillary and mandibulomaxillary facial angles at 11 + 0 to 13 + 6 weeks in fetuses with trisomy 18. Ultrasound Obstet. Gynecol..

[39] Borenstein M., Persico N., Dagklis T., Faros E., Nicolaides K.H. (2007). Frontomaxillary facial angle in fetuses with trisomy 13 at 11 + 0 to 13 + 6 weeks. Ulteasound Obstet. Gynecol..

[40] Lachmann R., Picciarelli G., Moratalla J., Greene N., Nicolaides K.H. (2010). Frontontomaxillary facial angle in fetuses with spina bifida at 11–13 weeks’ gestation. Ultrasound Obstet. Gynecol..

[41] Bernard J.P., Cuckle H.S., Stirnemann J.J., Salomone L.J., Ville Y. (2012). Screening for fetal spina bifida by ultrasound examination in the first trimester of pregnancy using fetal biparietal diameter. Am. J. Obstet. Gynecol..

[42] Czuba B., Cnota W., Wloch A., Wegrzyn P., Sodowski K., Wiglos M., Borowski D. (2013). Frontomaxillary facial angle measurement in screening for trisomy 18 at 11 + 0 to 13 + 6 weeks of pregnancy: A doubl-centre study. BioMed Res. Int..

[43] Malone F.D., Canick J.A., Ball R.H., Nyberg D.A., Comstock C.H., Bukowski R., Berkowitz R.L., Gross S.J., Dugoff L., Craigo S.D. (2005). First-trimester or second trimester screening, or both, for Down’s syndrome. N. Engl. J. Med..

[44] Reddy U.M., Filly R.A., Copel J.A. (2008). Prenatal imaging: Ultrasonography and magnetic resonance imaging. Obstet. Gynecol..

[45] Faiola S., Tsoi E., Huggon I.C., Allan L.D., Nicolaides K.H. (2005). Likelihood ratio for trisomy 21 in fetuses with tricuspid regurgitation at the 11 to 13 + 6-week scan. Ultasound Obstet. Gynecol..

[46] Kagan K.O., Gazzoni A., Sepulveda-Gonzalez G., Sotiriadis A., Nicolaides K.H. (2007). Discordance in nuchal translucency thickness in the prediction of severe twin-to-twin transfusion syndrome. Ultrasound Obstet. Gynecol..

[47] Maiz N., Valencia C., Emmanuel E.E., Staboulidou I., Nicolaides K.H. (2008). Screening for adverse pregnancy outcome by ducuts venosus Doppler at 11–13 + 6 weeks of gestation. Obstet. Gynecol..

[48] Bilardo C.M., Timmerman E., de Medina P.G., Clur A.S. (2011). Low-resistance hepatic artery flow in first-trimester fetuses: An ominous sign. Ultrasound Obstet. Gynecol..

[49] Nicolades K.H. (2011). Turning the pyramid of prenatal care. Fetal Diagn. Ther..

[50] Nicolaides K.H. (2011). Screening for fetal aneuploidies at 11 to 13 weeks. Prenat. Diagn..

[51] Bornestein E., Goncalves Rodriguez J.L., Alvarez Pavon E.C., Quiroga H., Or D., Divon M.Y. (2013). First-trimester sonographic findings associated with a Dandy-Walker malformation and inferior vermian hypoplasia. J. Ultrasound Med..

[52] McBrien A., Howley L., Yamamoto Y., Hutchinson D., Hirose A., Sekar P., Jain V., Motan T., Trines J., Savard W. (2013). Changes in fetal cardiac axis between 8 and 15 weeks’ gestation. Ultrasound Obstet. Gynecol..

[53] Wiechec M., Nocun A., Wiercinska E., Beithon J., Knafel A. (2014). First trimester tricuspid regurgitation and fetal abnormalities. J. Perinat. Med..

[54] Platt L.D. (2013). Should the first trimester ultrasound include anatomy survey?. Semin. Perinatol..

[55] Timor-Tritsch I.E., Fuchs K.M., Monteagudo A., D’Alton M.E. (2009). Performin a fetal anatomy scan at the time of first-trimester screening. Obstet. Gynecol..

[56] Rossi C., Prefumo F. (2013). Accuracy of ultrasonography at 11–14 weeks of gestation for detection of fetal structural anomalies: A systematic review. Obstet. Gynecol..

[57] Dukhovny S., Wilkins-Haug L., Shipp T.D., Benson C.B., Kaimal A.J., Reiss R. (2013). Absent fetal nasal bone: What does it mean for the euploid fetus?. J. Ultrasound Med..

[58] Pooh R.K., Kurjak A. (2014). Novel application of three-dimensional HDlive imaging in prenatal diagnosis from the first trimester. J. Perinat. Med..

[59] Saltvedt S., Almstrom A., Kublickas M., Valentin L., Grunewald C. (2006). Detection of malformationin chrmosomally normal fetuses by routine ultrasound at 12 or 18 weeks of gestation-a randomized controlled trial in 39,572 pregnancies. BJOG.

